# Advances in understanding grapevine downy mildew: From pathogen infection to disease management

**DOI:** 10.1111/mpp.13401

**Published:** 2023-11-22

**Authors:** Junbo Peng, Xuncheng Wang, Hui Wang, Xinghong Li, Qi Zhang, Meng Wang, Jiye Yan

**Affiliations:** ^1^ Beijing Key Laboratory of Environment Friendly Management on Fruit Diseases and Pests in North China Institute of Plant Protection, Beijing Academy of Agriculture and Forestry Sciences Beijing China

**Keywords:** effector biology, grapevine, *Plasmopara viticola*, sustainable control

## Abstract

*Plasmopara viticola* is geographically widespread in grapevine‐growing regions. Grapevine downy mildew disease, caused by this biotrophic pathogen, leads to considerable yield losses in viticulture annually. Because of the great significance of grapevine production and wine quality, research on this disease has been widely performed since its emergence in the 19th century. Here, we review and discuss recent understanding of this pathogen from multiple aspects, including its infection cycle, disease symptoms, genome decoding, effector biology, and management and control strategies. We highlight the identification and characterization of effector proteins with their biological roles in host–pathogen interaction, with a focus on sustainable control methods against *P. viticola*, especially the use of biocontrol agents and environmentally friendly compounds.

## Graphical abstract

A review and discussion of recent understanding of *Plasmopara viticola* from multiple aspects, including infection cycle, disease symptoms, genome decoding, effector biology, and management and control strategies.

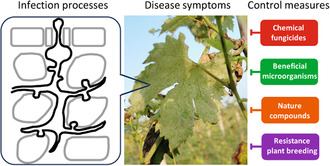

## 1 INTRODUCTION

Grapevine (*Vitis vinifera*) has established a deep connection with human culture in its long history spanning over 5000 years (Nascimento et al., 2019). Nowadays, grapevine is one of the most widely distributed fruit crops all around the world and comprises many varieties for wine production, table grapes and raisins for human consumption (Brilli et al., 2018; Xiang et al., 2021). Because of the huge market for these commodities, the grapevine industry is of great importance to economic expansion and increasing income in many countries and areas, with a global market size of over €29 billion (Nascimento et al., 2019).

Grapevines are susceptible to numerous pathogenic microorganisms, leading to various diseases. Downy mildew disease, caused by the obligately biotrophic peronosporomycete *Plasmopara viticola*, is one of the major threats in vineyards and causes huge losses in yield worldwide, especially in viticulture areas with relatively warm and humid climate conditions (Blasi et al., 2011; Yu et al., 2012). *P. viticola* was originally endemic on wild *Vitis* species of North America and was introduced into the Bordeaux area in 1871, probably with the acquisition of American grapevine rootstocks used as breeding stock for phylloxera resistance (Gessler et al., 2011; Liu, Weng, et al., 2019). Subsequently, *P. viticola* was detected in the Bordeaux area in 1878 and rapidly spread across Europe, leading to a severe pandemic throughout the continent in the following decade (Boso & Kassemeyer, 2008; Gessler et al., 2011).

In the field, *P. viticola* can infect all green tissues of grapevine, including leaves, inflorescences, fruit clusters, and young bunches, reducing the assimilation rate through a reduction in green leaf area and an influence on gas exchange in other green leaf tissues, resulting in significant losses in grapevine productivity and quality (Blasi et al., 2011; Moriondo et al., 2005; Yu et al., 2012). Typically, diseased leaves display yellow or reddish‐brown lesions on the upper surface, corresponding to white pathogen growth on the lower surface. Sometimes lesions are oily, somewhat angular and are located between the veins. The leaf lesions become brown and die with age (Musetti et al., 2005).

Because of grapevine's ability to be transformed and micropropagated via somatic embryogenesis, as well as its relatively small genome size relative to other perennials, this species has become a potential model organism for fruit crops in scientific research (Velasco et al., 2007). Additionally, *P. viticola* is considered a good candidate for the study of host adaptation of biotrophic pathogens (Dussert et al., 2016). In the past decade, hot issues of grapevine downy mildew and its cause, *P. viticola*, such as environmentally friendly control measures, pathogenesis and disease resistance, have received increasing attention and a great deal of progress has been made. In the current paper, we summarize and discuss the advances in understanding *P. viticola* from multiple aspects, including its infection cycle, effector biology and control measures.

## 2 INFECTION CYCLE

**FIGURE 1:**
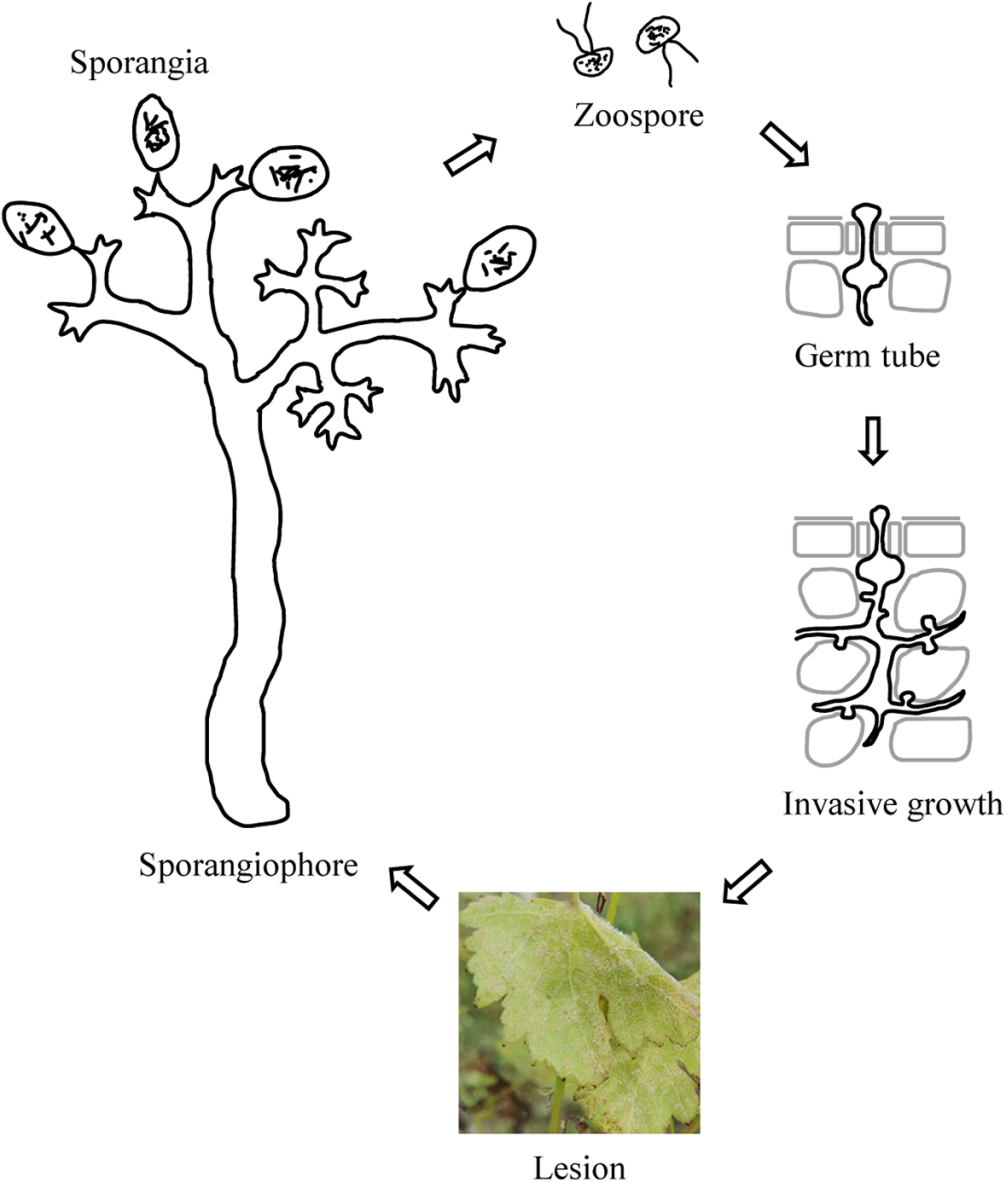
The infection cycle of *Plasmopara viticola*. Pathogen structures are shown in black.

The life cycle of *P. viticola* comprises an asexual multiplication phase that occurs during the plant vegetative period and a sexual phase that ensures the survival of the pathogen over winter (Díez‐Navajas et al., 2007). The primary sources of inoculum in spring derive from overwintering sexual oospores (Jürges et al., 2009; Vercesi et al., 1999). However, a rapid sequence of asexual propagation by sporangia under optimal conditions, such as high humidity and warm temperatures, causes severe epidemics and renders *P. viticola* a serious threat to viticulture (Jürges et al., 2009). The extremely efficient cycle of asexual propagation is responsible for the rapid spread of *P. viticola* worldwide (Islam et al., 2011). During the growing season, the asexually formed, lemon‐shaped sporangia release four to eight flagellate zoospores that swarm within the water film on the lower surface of the leaf (Jürges et al., 2009; Unger et al., 2007). On susceptible hosts, the motile zoospores are specially targeted to the stomata, where they shed their flagella and encyst (Jürges et al., 2009; Liu et al., 2015). The phenomenon of zoospores locating to the stomata is mediated by host cues (Islam et al., 2011; Kiefer et al., 2002). The encysted zoospores generate germ tubes that reach into the substomatal cavity, where they dilate into an infection vesicle (Kiefer et al., 2002; Liu et al., 2015; Yu et al., 2012). From the substomatal vesicle, a primary hypha emerges and develops into a mycelium that spreads inside the leaf tissue, extending mainly into the intercellular spaces of the spongy parenchyma and forming haustoria that penetrate the host cell wall (Jürges et al., 2009; Kiefer et al., 2002). Next, masses of hyaline sporangia are produced from sporangiophores at the lower leaf surface and are released and spread via wind currents or raindrops (Kortekamp, 2006). These sporangia start secondary infections as soon as weather conditions are favourable for their development and if protection is omitted (Kortekamp, 2006). At the end of autumn, numerous oospores, which represent the resting spores of *P. viticola*, form within fallen leaves and berries, allowing *P. viticola* to overwinter (Kortekamp, 2006). The life cycle of *P. viticola* is shown in Figure 1.

## 3 PATHOGENICITY GENES IN *P. VITICOLA*

The assembled genomes of several *P. viticola* isolates, including PvitFEM01 (NCBI SAMN06627059; Brilli et al., 2018), INRA‐PV221 (NCBI SAMN05415085; Dussert et al., 2019) and JL‐7‐2 (NCBI SAMN06231250; Yin, An, et al., 2017), are available in the National Center for Biotechnology Information (NCBI) database. The genome sizes of these isolates are 83.54, 92.94 and 101.3 Mb, containing 38,298, 15,960 and 17,014 protein‐coding genes, respectively. The differences may be caused by the large number of repetitive elements in their genomes, which could be the co‐evolutionary result of *P. viticola* and grapevine originating from different geographic regions. With respect to pathogenesis, only a couple of pathogenicity‐related genes, such as *PvCHS1* and *PvCHS2* (Werner et al., 2002), have been characterized, even though thousands of protein‐coding genes have been predicted (Dussert et al., 2019; Yin, An, et al., 2017). Therefore, the pathogenic mechanism of major *P. viticola* genes in susceptible genotypes is still poorly understood, which is partially attributed to the strictly biotrophic lifestyle of *P. viticola*, which makes this pathogen difficult to study in the laboratory (Chen et al., 2020; Liu, Lan, et al., 2018; Liu, Zhang, et al., 2018; Nascimento et al., 2019). Molecular research on the pathogen has therefore mainly focused on identifying secreted efforts during infection and deciphering the underlying mechanisms of grapevine–*P. viticola* interactions.

## 4 EFFECTOR BIOLOGY OF *P. VITICOLA*

Effectors are a large group of secreted pathogenicity‐related factors that can manipulate plant defence responses and modulate host cellular process to promote pathogen colonization in host plants (Lo Presti & Kahmann, 2017). Many effectors have been inferred to enter plant cells based on the physical interaction with host R proteins containing the nucleotide‐binding sites and leucine‐rich repeats (NB‐LRR) or the physiological effects, including activating programmed cell death or suppressing the activities of different cell death inducers when expressed intracellularly (Kale & Tyler, 2011). Effector proteins are classified into two groups based on their final destinations: cytoplasmic and apoplastic. The cytoplasmic effector proteins locate to different intracellular compartments and play various roles during pathogen host–interactions. The apoplastic effectors are well known to inhibit the activities of host‐secreted hydrolases or interrupt the functions of host receptors (Ma et al., 2017; Oh et al., 2009; Tian et al., 2004).

### 4.1 Cytoplasmic effectors

Cytoplasmic effectors belonging to different types have been identified in *P. viticola*; the most notable effectors are typically characterized as RxLR and CRN (crinkling and necrosis‐inducing or Crinkler) proteins (Jiang & Tyler, 2012; Yin, An, et al., 2017). Functional characterization of the two kinds of proteins has been widely performed in *P. viticola*.

#### 4.1.1 RxLR proteins

RxLR proteins are defined by a conserved N‐terminal motif similar in sequence, position and function to a host translocation signal RXLX(E/D/Q) present in the malaria parasite *Plasmodium falciparum* that enables delivery of effector proteins into human erythrocytes (Hiller et al., 2004; Schornack et al., 2010). Usually, the RxLR protein has an N‐terminal signal peptide, followed by an RxLR motif or its variant, and an EER motif.

In *P. viticola*, dozens of RxLR proteins have been functionally characterized in different isolates, including JL‐7‐2, ZJ‐1‐1 and CSIRO‐L‐2 (Yin et al., 2015), YL (Yin et al., 2019), PvitFEM01 (Brilli et al., 2018) and INRA‐PV221 (Dussert et al., 2016). Comparative analyses revealed that the number of RxLR proteins in *P. viticola* (Yin, An, et al., 2017), as well as *Hyaloperonospora arabidopsidis* (Baxter et al., 2010), *Plasmopara halstedii* (Sharma et al., 2015) and *Pseudoperonospora cubensis* (Savory et al., 2012), is less than in other plant‐pathogenic oomycetes, such as *Phytophthora infestans* (Haas et al., 2009), *Phytophthora ramorum* and *Phytophthora sojae* (Jiang et al., 2008; Tyler et al., 2006). The difference in RxLR protein number among these pathogens may be associated with their functional redundancy. Additionally, RxLR proteins appear to be absent from necrotrophic pathogens, such as *Pythium ultimum* (Lévesque et al., 2010), *Pythium insidiosum* (Adhikari et al., 2013; Krajaejun et al., 2011) and *Saprolegnia parasitica* (Jiang et al., 2013). The proliferation indicates *RxLR* genes have undergone a dramatic expansion in the *Phytophthora* and downy mildew lineage, which is thought to be a crucial innovation during the evolution of the biotrophic ancestor of *Phytophthora* spp. and the downy mildew lineage (Anderson et al., 2015). A high percentage of predicted RxLR proteins in *P. viticola* shows low similarity to RxLR proteins identified from other oomycetes, such as *H. arabidopsidis*, *P. infestans* and *P. sojae*, implying that RxLR proteins in *P. viticola* may have become more specific as a result of strong selection pressure during the evolution of *P. viticola* (Yin, Liu, et al., 2017).

**FIGURE 2:**
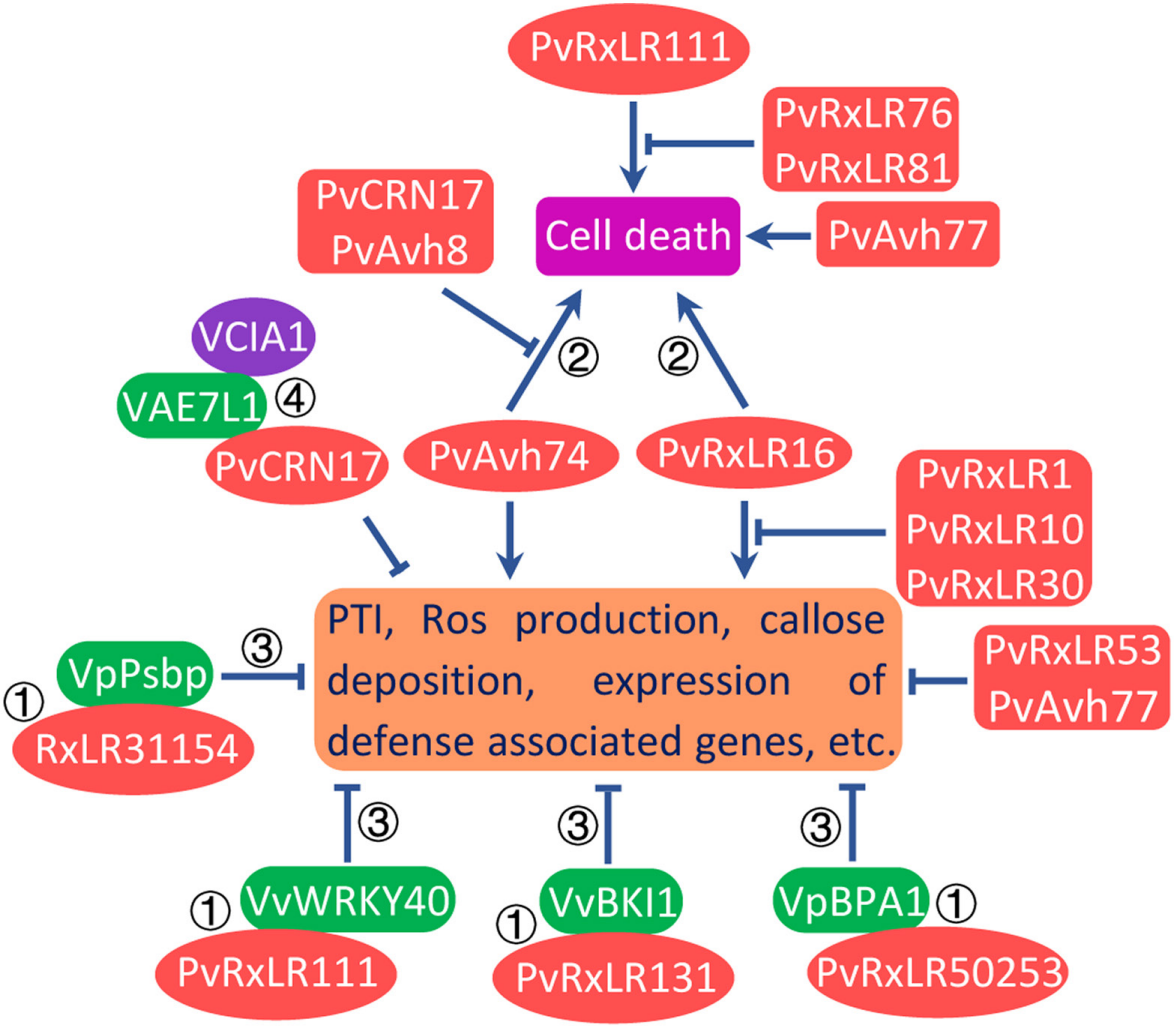
The regulatory pathway of RxLR and CRN proteins in *Plasmopara viticola*. ① RxLR proteins interact with or stabilize its target proteins. ② Cell death triggered by the proteins depends on SGT1, Hsp90, RAR1 and MAPK cascades. ③ Target proteins function as a negative regulator in plant immunity. ④ The PvCRN17 competes with VCIA1 to bind with VAE7L1 to suppress Fe‐S proteins‐mediated defence responses.

Functional analyses have also uncovered some common or specific characters of RxLR proteins in *P. viticola*. For example, suppression of plant immunity is the major activity of the RxLR secretome identified in *P. viticola* (Table 1), a feature shared by the RxLR secretome of *H. arabidopsidis* (Fabro et al., 2011; Pel et al., 2014). This is reasonable as the biotrophic oomycetes, including *P. viticola*, may have evolved to generate a relatively high percentage of RxLR effectors to suppress elicitin‐triggered cell death, keeping the host tissue alive for sustainable access to nutrients (Xiang et al., 2016). This inference was further exemplified by the inhibitory regulation among RxLR effectors shown in Figure 2. Moreover, the elicitor activity of RxLR proteins is associated with grapevine species, which can be supported by the fact that protein RxLR_PVITv1008311 without a signal peptide from *P. viticola* isolate PvitFEM01 elicits a cell death response in resistant *Vitis riparia* but not in susceptible grapevine *V. vinifera* (Brilli et al., 2018). The different responses may result from the differences in the recognition of *P. viticola* effectors between the two grapevines.

**TABLE 1:** The RxLR proteins characterized in *Plasmopara viticola.*

Protein name	Strain	Localization	Function	Reference
PvRxLR1	ZJ‐1‐1	Nucleus	Suppresses INF1‐ and BAX‐triggered cell death	Xiang et al. (2016), Yin et al. (2015)
PvRxLR2	ZJ‐1‐1	Nucleus and cytoplasm	Suppresses INF1‐ and BAX‐triggered cell death	Xiang et al. (2016)
PvRxLR3	JL‐7‐2	Nucleus and cytosol	Suppresses INF1‐ and BAX‐triggered cell death	Liu, Lan, et al. (2018)
PvRxLR4	JL‐7‐2, ZJ‐1‐1, CSIRO‐L‐2	—	Suppresses INF1‐ and BAX‐triggered cell death	Yin et al. (2015)
PvRxLR5	ZJ‐1‐1	Nucleus	Suppresses INF1‐ and BAX‐triggered cell death	Xiang et al. (2016), Yin et al. (2015)
PvRxLR6	JL‐7‐2	Nucleus and cytosol	Suppresses INF1‐ and BAX‐triggered cell death	Liu, Lan, et al. (2018)
PvRxLR7	JL‐7‐2	Nucleus and cytosol	Suppresses INF1‐ and BAX‐triggered cell death	Liu, Lan, et al. (2018)
PvRxLR8	JL‐7‐2	Nucleus	Suppresses INF1‐ and BAX‐triggered cell death	Liu, Lan, et al. (2018)
PvRxLR9 (PvAvh103)	ZJ‐1‐1, JL‐7‐2, CSIRO‐L‐2, YL	Nucleus and cytoplasm	Suppresses INF1‐ and BAX‐triggered cell death	Chen et al. (2020), Xiang et al. (2016), Yin et al. (2015)
PvRxLR10	ZJ‐1‐1	Nucleus and cytoplasm	Suppresses INF1‐ and BAX‐triggered cell death	Xiang et al. (2016), Yin et al. (2015)
PvRxLR11 (PvAvh8)	ZJ‐1‐1, JL‐7‐2, CSIRO‐L‐2, YL	Nucleus and cytoplasm	Suppresses INF1‐ and BAX‐triggered cell death	Chen et al. (2020), Xiang et al. (2016), Yin et al. (2015)
PvRxLR12	JL‐7‐2	—	Suppresses INF1‐ and BAX‐triggered cell death	Liu, Lan, et al. (2018)
PvRxLR13	JL‐7‐2, CSIRO‐L‐2	—	Suppresses INF1‐ and BAX‐triggered cell death	Yin et al. (2015)
PvRxLR14	JL‐7‐2	Nucleus	Suppresses INF1‐ and BAX‐triggered cell death	Liu, Lan, et al. (2018)
PvRxLR15	JL‐7‐2	Nucleus and cytosol	Suppresses INF1‐ and BAX‐triggered cell death	Liu, Lan, et al. (2018)
PvRxLR16	ZJ‐1‐1	Nucleus	Triggers cell death	Xiang et al. (2016)
PvRxLR17	ZJ‐1‐1	Nucleus and cytoplasm	Suppresses INF1‐ and BAX‐triggered cell death	Xiang et al. (2016)
PvRxLR18	JL‐7‐2	Nucleus and cytosol	Suppresses INF1‐ and BAX‐triggered cell death	Liu, Lan, et al. (2018)
PvRxLR19	ZJ‐1‐1	Nucleus and cytoplasm	Suppresses INF1‐ and BAX‐triggered cell death	Xiang et al. (2016), Yin et al. (2015)
PvRxLR20	JL‐7‐2	Nucleus and cytosol	Suppresses INF1‐ and BAX‐triggered cell death	Liu, Lan, et al. (2018), Yin et al. (2015)
PvRxLR21	JL‐7‐2	Nucleus and cytosol	Suppresses INF1‐ and BAX‐triggered cell death	Liu, Lan, et al. (2018)
PvRxLR22	ZJ‐1‐1	Nucleus	Suppresses INF1‐ and BAX‐triggered cell death	Xiang et al. (2016)
PvRxLR24	JL‐7‐2	Nucleus	Enhances cell death	Liu, Lan, et al. (2018)
PvRxLR25	ZJ‐1‐1	Nucleus and cytoplasm	Partially suppresses INF1‐ and BAX‐triggered cell death	Xiang et al. (2016)
PvRxLR27	ZJ‐1‐1	Nucleus and cytoplasm	Suppresses INF1‐ and BAX‐triggered cell death	Xiang et al. (2016)
PvRxLR28	ZJ‐1‐1	Nucleus and cytoplasm	Suppresses INF1‐ and BAX‐triggered cell death	Xiang et al. (2016)
PvRxLR29	ZJ‐1‐1	Nucleus and cytoplasm	Suppresses INF1‐ and BAX‐triggered cell death	Xiang et al. (2016)
PvRxLR30	ZJ‐1‐1	Nucleus	Suppresses INF1‐ and BAX‐triggered cell death	Xiang et al. (2016)
PvRxLR31	JL‐7‐2	Nucleus and cytosol	Does not suppress INF1‐triggered cell death	Liu, Lan, et al. (2018)
PvRxLR32	JL‐7‐2	—	Suppresses INF1‐ and BAX‐triggered cell death	Liu, Lan, et al. (2018)
PvRxLR35	JL‐7‐2	Nucleus	Enhances cell death	Liu, Lan, et al. (2018)
PvRxLR36	JL‐7‐2	Nucleus	Suppresses INF1‐ and BAX‐triggered cell death	Liu, Lan, et al. (2018)
PvRxLR37	JL‐7‐2	Nucleus and cytosol	Suppresses INF1‐ and BAX‐triggered cell death	Liu, Lan, et al. (2018)
PvRxLR38	JL‐7‐2	Nucleus and cytosol	Suppresses INF1‐ and BAX‐triggered cell death	Liu, Lan, et al. (2018)
PvRxLR39	JL‐7‐2	Nucleus	Suppresses INF1‐ and BAX‐triggered cell death	Liu, Lan, et al. (2018)
PvRxLR40	JL‐7‐2	Nucleus and cytosol	Suppresses INF1‐ and BAX‐triggered cell death	Liu, Lan, et al. (2018)
PvRxLR41	JL‐7‐2	Nucleus and cytosol	Enhances cell death	Liu, Lan, et al. (2018)
PvRxLR43	JL‐7‐2	Nucleus and cytosol	Suppresses INF1‐ and BAX‐triggered cell death	Liu, Lan, et al. (2018)
PvRxLR45	JL‐7‐2	Nucleus	Suppresses INF1‐ and BAX‐triggered cell death	Liu, Lan, et al. (2018)
PvRxLR47	JL‐7‐2	Plant cell membrane system	Suppresses INF1‐ and BAX‐triggered cell death	Liu, Lan, et al. (2018)
PvRxLR48	JL‐7‐2	Nucleus and cytosol	Suppresses INF1‐ and BAX‐triggered cell death	Liu, Lan, et al. (2018)
PvRxLR49	ZJ‐1‐1	Nucleus and cytoplasm	Suppresses INF1‐ and BAX‐triggered cell death	Xiang et al. (2016)
PvRxLR50	JL‐7‐2	Nucleus and cytosol	Suppresses INF1‐ and BAX‐triggered cell death	Liu, Lan, et al. (2018)
PvRxLR51	JL‐7‐2	Nucleus	Suppresses INF1‐ and BAX‐triggered cell death	Liu, Lan, et al. (2018)
PvRxLR53	JL‐7‐2	Nucleus and cytoplasm	Suppresses INF1‐triggered cell death	Liu et al. (2021)
PvRxLR54	JL‐7‐2	Chloroplasts and mitochondria	Suppresses INF1‐ and BAX‐triggered cell death	Liu, Lan, et al. (2018)
PvRxLR55	ZJ‐1‐1	Plasma membrane	Suppresses INF1‐ and BAX‐triggered cell death	Xiang et al. (2016)
PvRxLR57	JL‐7‐2	Nucleus and cytosol	Suppresses INF1‐ and BAX‐triggered cell death	Liu, Lan, et al. (2018)
PvRxLR61	JL‐7‐2	Chloroplasts and nucleus	Suppresses INF1‐ and BAX‐triggered cell death	Liu, Lan, et al. (2018)
PvRxLR63	ZJ‐1‐1	Nucleus	Partially suppresses INF1‐ and BAX‐triggered cell death	Xiang et al. (2016)
PvRxLR64	ZJ‐1‐1	Nucleus and cytoplasm	Suppresses INF1‐ and BAX‐triggered cell death	Xiang et al. (2016)
PvRxLR66	ZJ‐1‐1	Nucleus and cytoplasm	Suppresses INF1‐ and BAX‐triggered cell death	Xiang et al. (2016)
PvRxLR67	ZJ‐1‐1	Nucleus and cytoplasm	Partially suppresses INF1‐ and BAX‐triggered cell death	Xiang et al. (2016)
PvRxLR68	ZJ‐1‐1	Nucleus and cytoplasm	Suppresses INF1‐ and BAX‐triggered cell death	Xiang et al. (2016)
PvRxLR69	JL‐7‐2	Nucleus	Suppresses INF1‐ and BAX‐triggered cell death	Liu, Lan, et al. (2018)
PvRxLR70	JL‐7‐2	Nucleus	Suppresses INF1‐ and BAX‐triggered cell death	Liu, Lan, et al. (2018)
PvRxLR71	JL‐7‐2	—	Partially suppresses INF1‐ and BAX‐triggered cell death	Liu, Lan, et al. (2018)
PvRxLR73	JL‐7‐2	—	Suppresses INF1‐ and BAX‐triggered cell death	Liu, Lan, et al. (2018)
PvRxLR76	JL‐7‐2	Nucleus	Partially suppresses INF1‐ and BAX‐triggered cell death	Liu, Lan, et al. (2018)
PvRxLR77 (RxLR_PVITv1008311)	JL‐7‐2 (PvitFEM01)	—	Triggers the hypersensitive response in resistant cultivar *Vitis riparia*	Brilli et al. (2018), Xiang et al. (2021)
PvRxLR78	JL‐7‐2	—	Enhances cell death	Liu, Lan, et al. (2018)
PvRxLR80	JL‐7‐2	Endoplasmic reticulum	Does not suppress INF1‐triggered cell death	Liu, Lan, et al. (2018)
PvRxLR81	JL‐7‐2	Nucleus and cytosol	Partially suppresses INF1‐ and BAX‐triggered cell death	Liu, Lan, et al. (2018)
PvRxLR82	JL‐7‐2	Nucleus	Enhances cell death	Liu, Lan, et al. (2018)
PvRxLR83	JL‐7‐2	Nucleus and cytoplasm	Suppresses INF1‐ and BAX‐triggered cell death	Liu, Lan, et al. (2018)
PvRxLR85	JL‐7‐2	Plasma membrane	Partially suppresses INF1‐ and BAX‐triggered cell death	Liu, Lan, et al. (2018)
PvRxLR86	JL‐7‐2	Chloroplasts	Does not suppress INF1‐triggered cell death	Liu, Lan, et al. (2018)
PvRxLR89	JL‐7‐2	Nucleus and cytoplasm	Suppresses INF1‐ and BAX‐triggered cell death	Liu, Lan, et al. (2018)
PvRxLR90	JL‐7‐2	Plasma membrane	Suppresses INF1‐ and BAX‐triggered cell death	Liu, Lan, et al. (2018)
PvRxLR91	JL‐7‐2	Nucleus	Suppresses INF1‐ and BAX‐triggered cell death	Liu, Lan, et al. (2018)
PvRxLR93	JL‐7‐2	Nucleus	Suppresses INF1‐ and BAX‐triggered cell death	Liu, Lan, et al. (2018)
PvRxLR94	JL‐7‐2	Nucleus and cytoplasm	Suppresses INF1‐ and BAX‐triggered cell death	Liu, Lan, et al. (2018)
PvRxLR95	JL‐7‐2	Nucleus and cytoplasm	Enhances cell death	Liu, Lan, et al. (2018)
PvRxLR100	JL‐7‐2	Nucleus	Does not suppress INF1‐triggered cell death	Liu, Lan, et al. (2018)
PvRxLR101	JL‐7‐2	Nucleus	Partially suppresses INF1‐ and BAX‐triggered cell death	Liu, Lan, et al. (2018)
PvRxLR102	JL‐7‐2	Nucleus	Enhances cell death	Liu, Lan, et al. (2018)
PvRxLR103	JL‐7‐2	Nucleus	Enhances cell death	Liu, Lan, et al. (2018)
PvRxLR105	JL‐7‐2	Nucleus and cytoplasm	Does not suppress INF1‐triggered cell death	Liu, Lan, et al. (2018)
PvRxLR108	JL‐7‐2	Nucleus	Suppresses INF1‐ and BAX‐triggered cell death	Liu, Lan, et al. (2018)
PvRxLR111	JL‐7‐2	Nucleus (speckle‐like structures within the nucleus)	Enhances cell death	Liu, Lan, et al. (2018), Ma et al. (2021)
PvRxLR114	JL‐7‐2	—	Partially suppresses INF1‐ and BAX‐triggered cell death	Liu, Lan, et al. (2018)
PvRxLR115	JL‐7‐2	Nucleus and cytoplasm	Does not suppress INF1‐triggered cell death	Liu, Lan, et al. (2018)
PvRxLR117	JL‐7‐2	Nucleus	Enhances cell death	Liu, Lan, et al. (2018)
PvRxLR118	JL‐7‐2	Nucleus	Suppresses INF1‐ and BAX‐triggered cell death	Liu, Lan, et al. (2018)
PvRxLR120	JL‐7‐2	Nucleus	Suppresses INF1‐ and BAX‐triggered cell death	Liu, Lan, et al. (2018)
PvRxLR122	JL‐7‐2	Nucleus	Enhances cell death	Liu, Lan, et al. (2018)
PvRxLR123	JL‐7‐2	Nucleus and cytoplasm	Enhances cell death	Liu, Lan, et al. (2018)
PvRxLR124	JL‐7‐2	Nucleus and cytoplasm	Partially suppresses INF1‐ and BAX‐triggered cell death	Liu, Lan, et al. (2018)
PvRxLR126	JL‐7‐2	Plant cell membrane system	Suppresses INF1‐ and BAX‐triggered cell death	Liu, Lan, et al. (2018)
PvRxLR128	JL‐7‐2	Nucleus	Does not suppress INF1‐triggered cell death	Liu, Lan, et al. (2018)
PvRxLR131	JL‐7‐2	—	Suppresses INF1‐ and BAX‐triggered cell death	Lan et al. (2019)
PvRxLR134	JL‐7‐2	Nucleus	Suppresses INF1‐ and BAX‐triggered cell death	Liu, Lan, et al. (2018)
PvRxLR135	JL‐7‐2	Nucleus and cytoplasm	Suppresses INF1‐ and BAX‐triggered cell death	Liu, Lan, et al. (2018)
PvRxLR138	JL‐7‐2	Nucleus	Enhances cell death	Liu, Lan, et al. (2018)
PvRxLR142	JL‐7‐2	Nucleus	Suppresses INF1‐ and BAX‐triggered cell death	Liu, Lan, et al. (2018)
PvRxLR143	JL‐7‐2	Plasma membrane	Suppresses INF1‐ and BAX‐triggered cell death	Liu, Lan, et al. (2018)
PvRxLR144	JL‐7‐2	Nucleus and cytoplasm	Suppresses INF1‐ and BAX‐triggered cell death	Liu, Lan, et al. (2018)
PvRxLR146	JL‐7‐2	Nucleus and cytoplasm	Suppresses INF1‐ and BAX‐triggered cell death	Liu, Lan, et al. (2018)
PvRxLR147	JL‐7‐2	Nucleus and cytoplasm	Suppresses INF1‐ and BAX‐triggered cell death	Liu, Lan, et al. (2018)
PvRxLR149	JL‐7‐2	Nucleus and cytoplasm	Suppresses INF1‐ and BAX‐triggered cell death	Liu, Lan, et al. (2018)
PvRxLR150	JL‐7‐2	Nucleus and cytoplasm	Partially suppresses INF1‐ and BAX‐triggered cell death	Liu, Lan, et al. (2018)
PvRxLR151	JL‐7‐2	Endoplasmic reticulum	Suppresses INF1‐ and BAX‐triggered cell death	Liu, Lan, et al. (2018)
PvRxLR152	JL‐7‐2	Nucleus	Suppresses INF1‐ and BAX‐triggered cell death	Liu, Lan, et al. (2018)
PvRxLR153	JL‐7‐2	Plasma membrane	Suppresses INF1‐ and BAX‐triggered cell death	Liu, Lan, et al. (2018)
PvRxLR154	JL‐7‐2	Plasma membrane	Suppresses INF1‐ and BAX‐triggered cell death	Liu, Lan, et al. (2018)
PvRxLR158	JL‐7‐2	Nucleus and cytoplasm	Partially suppresses INF1‐ and BAX‐triggered cell death	Liu, Lan, et al. (2018)
PvRxLR159	JL‐7‐2	Nucleus and cytoplasm	Suppresses INF1‐ and BAX‐triggered cell death	Lei et al. (2019), Liu, Lan, et al. (2018)
PvRxLR160	JL‐7‐2	—	Suppresses INF1‐ and BAX‐triggered cell death	Liu, Lan, et al. (2018)
PvRxLR161	JL‐7‐2	Chloroplasts and nucleus	Suppresses INF1‐ and BAX‐triggered cell death	Liu, Lan, et al. (2018)
PvRxLR50253	YL	Plasma membrane, cytoplasm, and nucleus	Suppresses INF1‐ and BAX‐triggered cell death	Yin et al. (2022)
PvAvh74	YL	Nucleus	Triggers cell death	Yin et al. (2019)
PvAvh77	YL	Nucleus	Triggers cell death	Fu et al. (2023)

*Note*: The symbol ‘—’ indicates the localization of these RxLR proteins was undetermined.

Although a number of RxLR effectors have been identified, the regulatory mechanisms between RxLR effectors with their interactive targets from plant cells have only been investigated for a couple of effectors. RxLR effectors PvRxLR111 with PvRxLR50253 target and stabilize grapevine proteins VvWRKY40 with VpBPA1 to suppress plant immunity through decreasing H_2_O_2_ accumulation and promote pathogen infection (Ma et al., 2021; Yin et al., 2022). Another RxLR protein PvRxLR131 targets *V. vinifera* BRI1 kinase inhibitor 1 (VvBKI1) in the plasma membrane as a strategy for promoting infection (Lan et al., 2019). However, it is challenging to determine whether the RxLR proteins have virulence functions in susceptible grape cultivars and characterize their correlations with correspondent resistance (R) proteins, even though the gene‐for‐gene relationship between an avirulent *RxLR* gene with its cognate resistance (R) gene has been well described in other pathogenic oomycetes, such as *P. sojae* Avr1a and Avr3a (Qutob et al., 2009), Avr1b‐1 and Avr1k/Rps1k (Song et al., 2013), Avr3b (Dong et al., 2011), Avr3c (Dong et al., 2009), Avr4/Rps4 (Dou et al., 2010), Avr6/Rps6 (Dou et al., 2010), *H. arabidopsidis* ATR13/RPP13 (Allen et al., 2004), ATR1^NdWsB^/RPP1 (Rehmany et al., 2005), ATR39/RPP39 (Goritschnig et al., 2012) and *P. infestans* Avr1/R1 (Du et al., 2015), Avr2/R2 (Gilroy et al., 2011), Avr4/R4 (van Poppel et al., 2008), Avr‐blb1/Rpi‐blb1 (Vleeshouwers et al., 2008) and AVRblb2/Rpi‐blb2 (Oh et al., 2009).

#### 4.1.2 CRN proteins

CRN proteins are small, secreted proteins first identified in *P. infestans* and described as causing a crinkling and necrosis phenotype when ectopically expressed in planta (Torto et al., 2003; Xiang et al., 2021). CRN proteins have a conserved N‐terminal LXLFLAK motif and a conserved HVLVXXP motif followed by variable carboxyl (C)‐terminal sequences (Haas et al., 2009; Xiang et al., 2021).

CRN proteins are conserved and ubiquitously present in all sequenced plant‐pathogenic oomycete species, including *Saprolegniales* (Gaulin et al., 2008; Schornack et al., 2010), *Pythiales* (Cheung et al., 2008; Lévesque et al., 2010), *Albuginales* (Kemen et al., 2011; Links et al., 2011) and *Peronosporales* (Mafurah et al., 2015; Rajput et al., 2015), in contrast to the RxLR effectors that have only been identified in *Peronosporales* and *Albuginales* (Yin et al., 2015). The difference suggests that the CRN protein family may have arisen before the emergence of haustoria and disseminated into other microorganisms by horizontal gene transfer, whereas the RxLR effectors emerged and diversified in accordance with the evolution of haustoria (Schornack et al., 2010; Sun et al., 2011; Yin et al., 2015). Recent data have revealed that CRN proteins are present in other pathogenic and free‐living eukaryotes, including *Batrachochytrium dendrobatidis* (Sun et al., 2011), *Batrachochytrium salamandrivorans* (Farrer et al., 2017), *Rhizophagus irregularis* (Voß et al., 2018), members of *Viridiplantae* and amoebozoans (Zhang et al., 2016), suggesting that CRN proteins may be more ubiquitously distributed than predicted. However, CRNs are absent in animal‐pathogenic oomycetes, suggesting that the evolution and occurrence of this kind of protein may be associated with virulence and adaption on susceptible plants (Gaulin et al., 2018; Voß et al., 2018).

In terms of *P. viticola*, an array of *CRN‐like* genes have been cloned and characterized from isolates JL‐7‐2 (Yin, An, et al., 2017), PvitFEM01 (Brilli et al., 2018) and YL (Xiang et al., 2021). Sequence alignments revealed that 27 *PvCRN* genes from isolate YL share high similarities in nucleotides with their orthologues from another three *P. viticola* isolates, JL‐7‐2, INRA‐PV221 and PVitFEM01. The high level of intraspecific nucleotide polymorphism among the *CRN*‐*like* genes from these four *P. viticola* isolates is considered to be a reflection of pathogen evolution adaptation to different grapevine genotypes in distinct geographic areas (Xiang et al., 2021). Moreover, it was found that gene duplication (*PvCRN27* and *PvCRN29*, *PvCRN10* and *PvCRN11*) and fragment recombination of *CRN*s occurred during adaptive evolution in *P. viticola*, which is analogous to the *CRN* genes in *P. sojae* (Shen et al., 2013) and *P. infestans* (Haas et al., 2009). Gene recombination was mainly generated with three different patterns. In the first pattern, *CRN* genes contain a highly conserved N‐terminal sequence, but differ in the C‐terminal sequence; these genes include *PvCRN31* and *PvCRN11*, *PvCRN21* and *PvCRN22*, *PvCRN12*, *PvCRN35* and *PvCRN17*. The second recombination pattern is that *CRN* genes have a highly conserved C‐terminal sequence but display diversity in the N‐terminal coding sequence, such as *PvCRN1*, *PvCRN4* and *PvCRN30*. Finally, a novel *CRN* gene is composed of distinct fragments from at least two other *PvCRN* genes, which can be evidenced by the genes *PvCRN15* and *PvCRN16* (Xiang et al., 2021). To some extent, this phenomenon may explain the CRN family expansion and sequence divergence of the three oomycetes when compared to other fungi and oomycetes.

Although the first CRN protein was characterized as a crinkling and necrosis‐inducing factor on expression in planta, a characteristic of plant innate immunity (Haas et al., 2009; Torto et al., 2003), an array of studies has revealed that this is not a common feature for CRN proteins or their C‐terminal domain, and one set of CRN proteins even displays the opposite function. For example, PsCRN63 (Liu, Ye, et al., 2011), PcCRN4 (Mafurah et al., 2015), PiCRN8 (van Damme et al., 2012) and PcCRN83_152 (Amaro et al., 2018) induce cell death, but some other CRN effectors, such as VmEP1 (Li et al., 2015), PsCRN115 (Liu, Ye, et al., 2011), PsCRN70 (Rajput et al., 2014) and PsCRN161 (Rajput et al., 2015), suppress cell death triggered by other elicitins, indicating that CRN proteins possess diverse functions beyond cell death induction (Amaro et al., 2018; Stam et al., 2013; Voß et al., 2018). In *P. viticola*, most characterized PvCRN proteins suppress or attenuate cell death triggered by other elicitins when transiently expressed in *Nicotiana benthamiana* (Table 2), which is also inconsistent with the initially documented roles of CRN proteins. Additionally, although many *PvCRN* genes have been identified in *P. viticola*, the molecular functions have been explained for only a couple of *PvCRN* genes. For example, the CRN protein PvCRN17 competed with VCIA1 to bind with VAE7L1, demolishing the cytosolic iron–sulphur (Fe‐S) cluster assembly (CIA) Fe‐S cluster transfer complex to suppress Fe‐S protein‐mediated defence responses (Figure 2). In future, the most important and urgent task is to identify the host targets of PvCRN effectors and investigate their molecular functions, which drives the identification of unknown components in plant immunity and metabolism, as well as promoting biotechnology innovations.

**TABLE 2:** The CRN proteins characterized in *Plasmopara viticola*.

Protein name	Strain	Localization	Function	Reference
PvCRN1	YL	Plasma membrane, cytoplasm, and nucleus	Neither suppresses INF1‐ and BAX‐triggered cell death nor induces cell death	Xiang et al. (2021)
PvCRN2	YL	Plasma membrane, cytoplasm, and nucleus	Partially suppresses or delays INF1‐ but not BAX‐triggered cell death	Xiang et al. (2021)
PvCRN4	YL	Plasma membrane, cytoplasm, and nucleus	Neither suppresses INF1‐ and BAX‐triggered cell death nor induces cell death	Xiang et al. (2021)
PvCRN6	YL	Plasma membrane, cytoplasm, and nucleus	Neither suppresses INF1‐ and BAX‐triggered cell death nor induces cell death	Xiang et al. (2021)
PvCRN7	YL	Plasma membrane, cytoplasm, and nucleus	Neither suppress INF1‐ and BAX‐triggered cell death nor induces cell death	Xiang et al. (2021)
PvCRN9	YL	Plasma membrane, cytoplasm, and nucleus	Neither suppresses INF1‐ and BAX‐triggered cell death nor induces cell death	Xiang et al. (2021)
PvCRN10	YL	Plasma membrane, cytoplasm, and nucleus	Suppresses BAX‐ but not INF1‐triggered cell death	Xiang et al. (2021)
PvCRN11	YL	Plasma membrane, cytoplasm, and nucleus	Induces spotted necrosis	Xiang et al. (2021)
PvCRN12	YL	Plasma membrane, cytoplasm, and nucleus	Suppresses BAX‐ but not INF1‐triggered cell death	Xiang et al. (2021)
PvCRN14	YL	Plasma membrane, cytoplasm, and nucleus	Suppresses BAX‐ but not INF1‐triggered cell death	Xiang et al. (2021)
PvCRN15	YL	Plasma membrane and the nuclear envelope	Suppresses BAX‐ but not INF1‐triggered cell death	Xiang et al. (2021)
PvCRN16	YL	Plasma membrane	Attenuates cell death triggered by BAX and INF1	Xiang et al. (2021)
PvCRN17	YL	Mainly localized in the plasma membrane and nucleus	Attenuates cell death triggered by BAX and INF1	Xiang et al. (2021)
PvCRN18	YL	Plasma membrane, cytoplasm, and nucleus	Suppresses BAX‐ but not INF1‐triggered cell death	Xiang et al. (2021)
PvCRN19	YL	Nucleus	Neither suppresses INF1‐ and BAX‐triggered cell death nor induces cell death	Xiang et al. (2021)
PvCRN20	YL	Plasma membrane, cytoplasm, and nucleus	Suppress INF1‐and BAX‐triggered cell death	Xiang et al. (2021)
PvCRN21	YL	Plasma membrane, cytoplasm, and nucleus	Neither suppresses INF1‐ and BAX‐triggered cell death nor induces cell death	Xiang et al. (2021)
PvCRN22	YL	Plasma membrane, cytoplasm, and nucleus	Suppresses BAX‐ but not INF1‐triggered cell death	Xiang et al. (2021)
PvCRN23	YL	Plasma membrane, cytoplasm, and nucleus	Suppresses BAX‐ but not INF1‐triggered cell death	Xiang et al. (2021)
PvCRN24	YL	Plasma membrane, cytoplasm, and nucleus	Suppresses BAX‐ but not INF1‐triggered cell death	Xiang et al. (2021)
PvCRN25	YL	Plasma membrane, cytoplasm, and nucleus	Suppresses BAX‐ but not INF1‐triggered cell death	Xiang et al. (2021)
PvCRN26	YL	Plasma membrane, cytoplasm, and nucleus	Suppresses BAX‐ but not INF1‐triggered cell death	Xiang et al. (2021)
PvCRN27	YL	Nucleus	Neither suppresses INF1‐ and BAX‐triggered cell death nor induces cell death	Xiang et al. (2021)
PvCRN29	YL	Nucleus	Neither suppresses INF1‐ and BAX‐triggered cell death nor induces cell death	Xiang et al. (2021)
PvCRN30	YL	Plasma membrane	Suppresses BAX‐ but not INF1‐triggered cell death	Xiang et al. (2021)
PvCRN31	YL	Plasma membrane, cytoplasm, and nucleus	Neither suppresses INF1‐ and BAX‐triggered cell death nor induces cell death	Xiang et al. (2021)
PvCRN35	YL	Plasma membrane and the nuclear envelope	Suppresses BAX‐ but not INF1‐triggered cell death	Xiang et al. (2021)

#### 4.1.3 YxSL[RK] proteins

Besides RxLR and CRN motifs, YxSL[RK] is another conserved motif that has been identified in the secreted and non‐secreted proteins of oomycete species including *P. ultimum* (Lévesque et al., 2010), *P. infestans* and *P. sojae* (Adhikari et al., 2013), and *S. parasitica* (Jiang et al., 2013). The YxSL[RK] motif shares similarity in sequence and position with the canonical RxLR motif and appears to be a signature for a novel family of secreted proteins that function as effectors (Adhikari et al., 2013; Lévesque et al., 2010). However, whether the YxSL[RK] motif defines a host‐translocation domain as noted for RxLR effectors remains to be determined (Lévesque et al., 2010). *P. viticola* contains a relatively high number (25) of putative secreted YxSLK[RK] proteins compared to the other biotrophic oomycetes *P. halstedii* (16), *H. arabidopsidis* (14) and *Albugo laibachii* (9), but a much lower number than *Phytophthora* species, including *P. infestans* (43), *P. capsici* (45) and *P. sojae* (61) (Brilli et al., 2018). However, the molecular roles and underlying mechanisms of this kind of protein have not yet been revealed.

### 4.2 Apoplastic effectors

Oomycetes not only secrete large numbers of typical RxLR and CRN effectors targeted to the host cytoplasm to alter host physiology and facilitate pathogen colonization, they also release an extensive range of apoplastic effectors that interact with extracellular targets and surface receptors to facilitate infection (Jiang & Tyler, 2012; Yin et al., 2015). The genome of sequenced oomycetes has revealed large complex families of apoplastic effectors, including secreted hydrolytic enzymes such as lyases, proteases, lipases and glycosylases that probably degrade plant tissue, enzyme inhibitors to protect against host defence enzymes, necrotizing toxins such as necrosis‐ and ethylene‐inducing peptide‐like proteins (NLPs), *Phytophthora cactorum* factors and secreted cysteine‐rich proteins, that are implicated in pathogenesis during symptom development (Haas et al., 2009; Jiang & Tyler, 2012; Tyler et al., 2006).

The first characterized member of NLP family, the Nep1 protein, was isolated from culture filtrates of *Fusarium oxysporum* (Bailey, 1995). Experimental tests demonstrated that Nep1 was capable of inducing ethylene biosynthesis as well as necrosis in a wide variety of Dicotyledoneae but not in Monocotyledoneae (Bailey, 1995). Since then, more NLPs have been identified in various phytopathogenic microorganisms, including fungi, bacteria and oomycetes (Xiang et al., 2022). According to the induced phenotypes, NLPs can be classified into two groups: the cytotoxic NLPs, which are able to permeabilize the cellular membrane of dicotyledonous plants and cause necrosis as well as a myriad of other defence responses (Seidl & Van den Ackerveken, 2019), or the noncytotoxic NLPs, with the ability to activate cell death‐independent immunity (Seidl & Van den Ackerveken, 2019; Xiang et al., 2022). It was assumed that obligately biotrophic pathogens generally contained the noncytotoxic NLPs, as the biotrophs rely on living plant tissues for their growth and reproduction (Schumacher et al., 2020; Seidl & Van den Ackerveken, 2019). However, functional analyses of this kind of protein from obligate biotrophs were only performed on a couple of NLPs in *P. viticola* (Askani et al., 2021; Schumacher et al., 2020; Xiang et al., 2022) and *H. arabidopsidis* (Cabral et al., 2012). For example, a few *NLP* genes have been identified in *P. viticola* and most were highly expressed during the early stages of infection, suggesting that these genes may play major roles during pathogen penetration or initial colonization inside host tissues (Askani et al., 2021; Schumacher et al., 2020; Xiang et al., 2022). However, whether *PvNLP* genes contribute to virulence for *P. viticola* is still unknown. Several tested NLPs (PvNLP1–8) are known to be unable to cause necrosis in *N. benthamiana* (Askani et al., 2021; Schumacher et al., 2020), which is in line with the noncytotoxic effect of tested NLPs of *H. arabidopsidis* (Cabral et al., 2012). Conversely, Xiang et al. (2022) recently reported that PvNLP7 was able to cause necrosis and enhance *P. capsici* resistance in *N. benthamiana* with *H. arabidopsidis* resistance in *Arabidopsis*. Further research is necessary to resolve these controversial issues. Additionally, even though the major NLPs identified in *P. viticola* displayed noncytotoxic phenotypes, some of the NLPs suppressed plant growth and enhanced plant resistance against downy mildew, which implies that these NLPs may play roles in different ways independent of necrosis.

### 4.3 RNA

Apart from effector proteins, bidirectional cross‐species small RNA (sRNA)‐mediated gene regulation during the compatible interaction has also been revealed in *P. viticola*. The sRNAs generated by *P. viticola* trigger the cleavage of grapevine genes and, vice versa, the sRNAs processed from grapevine transcripts target *P. viticola* messenger RNAs (Brilli et al., 2018). The shuffling of low molecular weight RNA between *P. viticola* with its host implies that bidirectional communication of sRNAs is an important invasion or resistance strategy adopted by both organisms during the infection. However, the bidirectional exchange pathway and mechanism of sRNAs have not yet been revealed.

## 5 MANAGEMENT AND CONTROL STRATEGIES

In the history of grapevine downy mildew disease management, an array of commodities aimed at killing the pathogen directly or activating induced system resistance indirectly has been developed and widely used in the field. Based on their composition and physicochemical properties, these commodities can be classified into two groups: chemical fungicides and biological control agents. Alternative measures aimed to reduce the use of chemical fungicides but retain good control over the causal agent, including breeding disease‐resistant grapevine varieties and the use of resistance inducers, have received more attention during recent years. Here, we briefly summarize the main developments in these management strategies and discuss their advantages and disadvantages in practical usage.

### 5.1 Chemical fungicides

**FIGURE 3:**
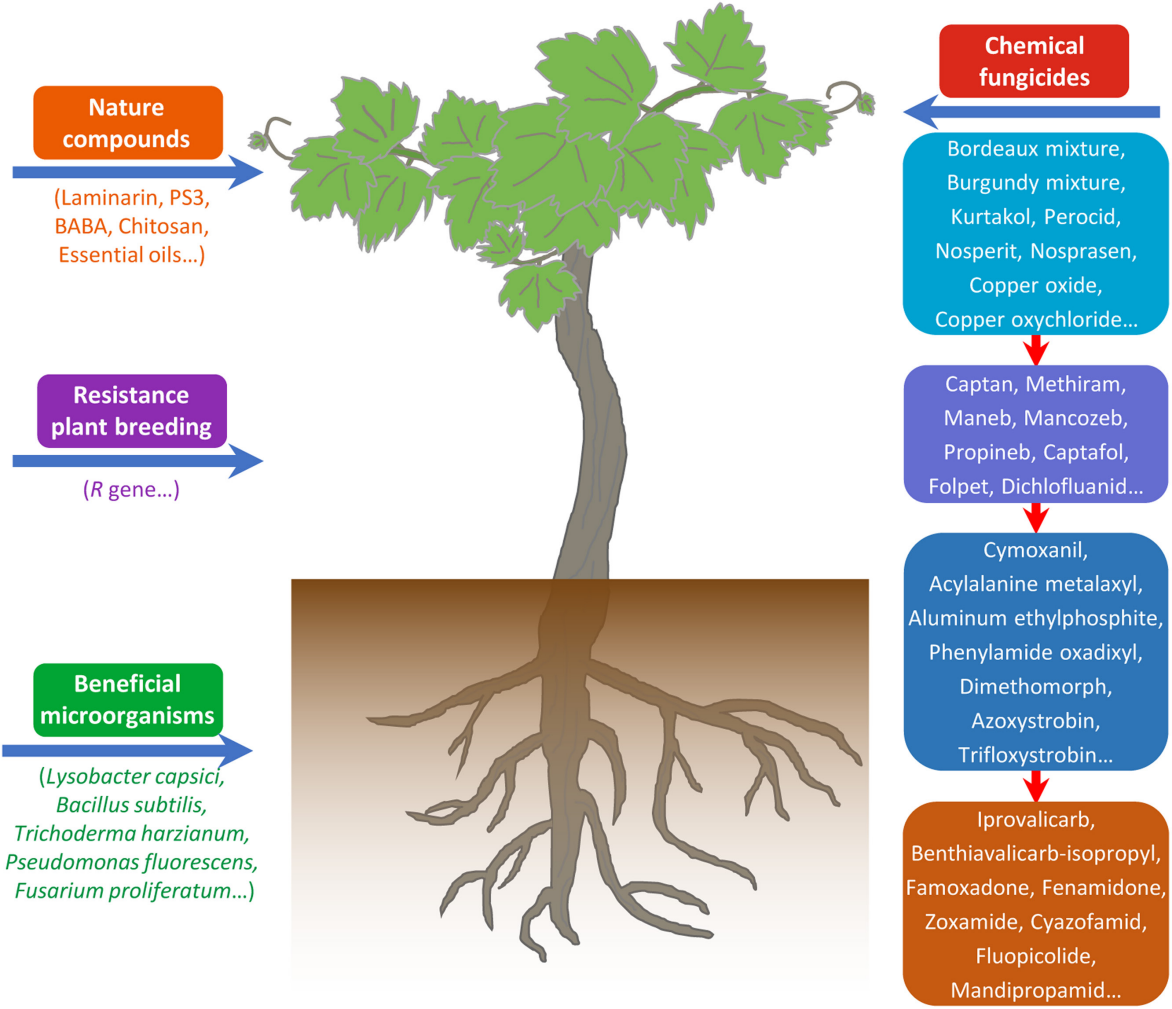
Different methods used to control *Plasmopara viticola*.

In organic viticulture, chemical control is the most effective method currently used to control grapevine downy mildew (Battiston et al., 2018; Selim, 2013). In the history of grapevine downy mildew control, the copper sulphate‐based Bordeaux mixture represents the first milestone and it is considered to be the first oomycete fungicide obtained during the development history of phytomedicine (Millardet, 1885; Selim, 2013). Afterwards, a series of copper or sulphate compounds, including Burgundy mixture (Masson, 1887), kurtakol (Lustner, 1922), copper salt of oxyquinoline (Meyer, 1932) and copper oxide (Osterwalder, 1939), were invented and applied to control *P. viticola*. Thanks to the development of new stable compounds, reduced costs and decreased phytotoxicity, many acupric fungicides, including captan, methiram, maneb, mancozeb, propineb, captafol, folpet and dichlofluanid, have been developed and their use has become prevalent among grapevine growers (Gessler et al., 2011). After the 1970s, systemic fungicides, including cymoxanil (Serres & Carraro, 1976), acylalanine metalaxyl (Vial et al., 1978; Wicks, 1980), aluminium ethylphosphite or fosetyl‐Al (Boubals et al., 1979), phenylamide oxadixyl (Gisi et al., 1983), dimethomorph (Wicks & Hall, 1990) and azoxystrobin (Bugaret et al., 1998), were greeted with enthusiasm in the market, partly because of their resistance to rainfall wash‐off and their outstanding curative effects against established infections (Boubals & Lafon, 1981; Gessler et al., 2011). In the past two decades, many new active ingredients, including famoxadone (Andrieu et al., 2000), benthiavalicarb‐isopropyl (Reuveni, 2003), fluopicolide (Gouot, 2006) and mandipropamid (Lamberth et al., 2006), have been developed and applied because of their high efficiency against downy mildew and favourable toxicological features (Figure 3).

Because of health and environmental concerns, as well as the detrimental effect on wine quality of long‐term use of copper‐based fungicides, the usage of cupric fungicides is currently restricted by European Union Regulation 473/2002 and copper‐based formulations used in organic farming are limited to 6 kg/ha per year in most European countries (Garde‐Cerdán et al., 2017; Kortekamp, 2006). The development of novel copper‐based formulations appears to be a promising approach to enhance control efficiency and minimize the side effects caused by copper (Battiston et al., 2019; La Torre et al., 2010). The advent of nanotechnology provides an innovative perspective to develop pesticides that share slow‐release systems to optimize their distribution and persistence, therefore enhancing the protective effect and control efficiency. For example, Cu(II) compounds formulated with synthetic nanostructured hydroxyapatite have resulted in reduced disease severity and higher efficacy even under rain‐washed conditions (Battiston et al., 2018, 2019). Although promising results were achieved by the engineered nanoparticles, it is necessary to evaluate the cytotoxicity and genotoxicity of such particles within the plant tissues, as some reports have claimed adverse effects of these nanoparticles on the growth and development of tested plants (Lee et al., 2008; Lin & Xing, 2008). Besides new formulations, the development of alternative strategies to reduce the use of classic chemical fungicides for grapevine downy mildew protection also seems to be an urgent and promising task.

### 5.2 Beneficial microorganisms

Plant resistance can be triggered or strengthened after the recognition of pathogenic or beneficial microbes through pathogen‐ or microbe‐associated molecular patterns by host‐specific receptors (Lakkis et al., 2019). Several beneficial microorganisms, including *Lysobacter capsici* (Puopolo et al., 2013; Segarra et al., 2015), *Bacillus subtilis* (Li et al., 2019; Shen et al., 2016), *Trichoderma harzianum* (Perazzolli et al., 2012; Roatti et al., 2013), *Pseudomonas fluorescens* (Lakkis et al., 2019; Shoresh et al., 2010) and *Fusarium proliferatum* (Bakshi et al., 2001; Perazzolli et al., 2012), have been developed as attractive candidates in the biological control of *P. viticola* (Figure 3). Among them, *B. subtilis* is one of the most commercialized biological control agents (Li et al., 2019). These biocontrol agents mediate plant resistance by producing various bioactive compounds, such as fengycin and surfactin (Li et al., 2019), khatmiamycin (Abdalla et al., 2011), staurosporine (Islam et al., 2011), banchromene (Tatong et al., 2014), cryptosporiopsin A, hydroxypropan‐2′,3′‐diol orsellinate and cyclic pentapeptide (Talontsi et al., 2012), oligomycins and pamamycin homologues (Dame et al., 2016), to inhibit *P. viticola* directly or activate induced systemic resistance, which is associated with priming phytohormone (salicylic acid, 1‐aminocyclopropane‐1‐carboxylic acid, abscisic acid) production, stilbenic phytoalexin and callose accumulation, and expression of defence‐related genes (Lakkis et al., 2019; Perazzolli et al., 2012; Roatti et al., 2013).

Although many biocontrol microorganisms display a control effect against downy mildew under experimental conditions, the use of these biocontrol agents in agriculture is still far from widespread because of the unmanageable and changeable abiotic stresses (Roatti et al., 2013). Various factors, including environmental factors, production cost, the time period that microorganisms can be stored in packaging, their survival and activity on the plant and in soils, can comprehensively impact the control efficiency (Perazzolli et al., 2012; Roatti et al., 2013; Segarra et al., 2015). A growing amount of research has revealed that appropriate formulation helps to enhance the efficiency of biocontrol agents. For example, application of *L. capsici* AZ78 in combination with a low dose of a copper‐based fungicide leads to higher control efficiency against grapevine downy mildew (Puopolo et al., 2013). Use of *L. capsici* AZ78 together with corn steep liquor, lignosulfonate and polyethylene glycol in the formulation improves the survival of *L. capsici* AZ78 cells by one order of magnitude and ensures a high level of protective efficacy (Segarra et al., 2015). *T. harzianum* T39‐induced resistance is attenuated by the combined abiotic stress of heat and drought (Roatti et al., 2013), therefore the optimized formulation is a crucial step in biopesticide development and is an efficient way to maintain persistence in terms of biological control under field conditions.

### 5.3 Pathogen‐resistant grapevine breeding

**FIGURE 4:**
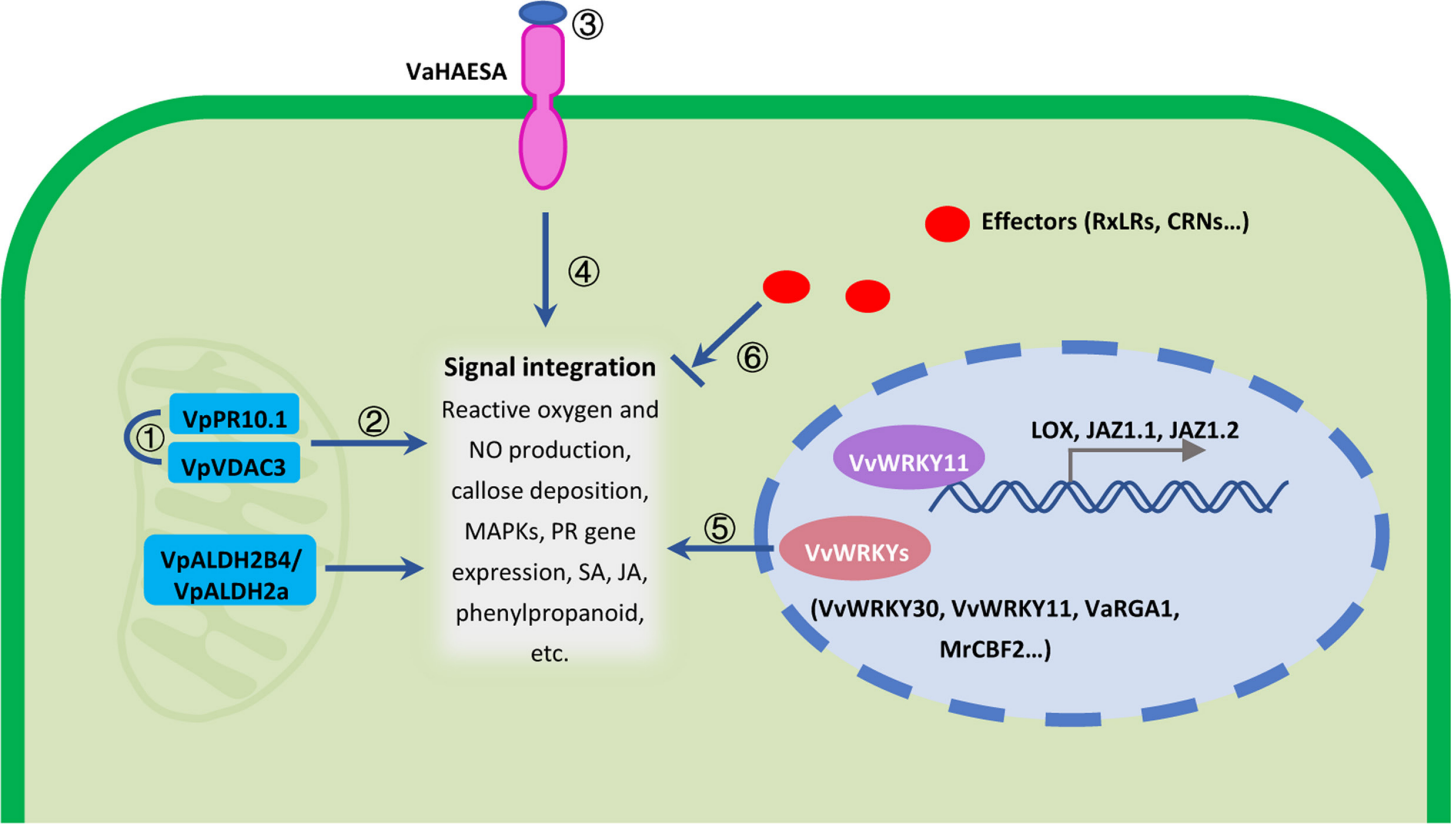
The resistant responses of grapevine against *Plasmopara viticola*. ① VpPR10.1 interacts with VpVDAC3. ② VpPR10.1 and VpVDAC3 induce the expression of *NbAOX*, *NbRbohB* and *NbAPX*. ③ PAMPs recognized by the membrane‐localized LRR‐RLK protein VaHAESA. ④ VaHAESA triggers the downstream PAMP‐triggered immunity response. ⑤ WRKY transcription factors induce defence responses, including expression of *PR* genes, accumulation of jasmonic acid (JA) or salicylic acid (SA). ⑥ Effectors secreted by pathogens suppress the defence response.

Grapevine distributed in different geographic areas exhibited susceptibility and resistance against *P. viticola* at various levels. Generally, the level of grapevine resistance to *P. viticola* is divided into five classes: immune, extremely resistant, resistant, partly resistant and susceptible (Yu et al., 2012). All the traditional cultivars of *V. vinifera*, which is the most widely cultivated grapevine species and suitable for wine and table grape production, are susceptible to downy mildew, although variations of susceptibility are observed among different cultivars or even between clones of the same variety (Blanc et al., 2012; Blasi et al., 2011; Boso et al., 2014). In contrast, the North American and Asian *Vitis* species belonging to the *Euvitis* subgenus or *Muscadinia* subgenus exhibit variable levels of resistance to *P. viticola*, ranging from moderate resistance, such as *V. rupestris*, to high resistance, including *V. rubra*, *V. candicans*, *V. amurensis*, *V. riparia*, *V. cinerea* and *Muscadinia rotundifolia* (Blasi et al., 2011; Gessler et al., 2011). In nature, control of downy mildew on these traditional grapevine varieties generally relies on the massive use of pesticides (Peressotti et al., 2010). However, routine use of fungicides is becoming increasingly restrictive because of their heavy cost to grapevine production, high risk to human health and adverse impacts on environment (Blanc et al., 2012; Peressotti et al., 2010). Moreover, a growing number of fungicide‐resistant *P. viticola* strains have been detected in the vineyard, reducing the efficiency of fungicide application (Blanc et al., 2012). Therefore, the search for alternative methods to control the disease is important for viticulture (Peressotti et al., 2010). In this context, plant breeding for disease resistance based on the introgression of resistance traits from ancestral species into domesticated varieties appears to be an attractive and environmentally friendly way to control grapevine downy mildew (Blanc et al., 2012; Vezzulli et al., 2019). During the last 20 years, research on the genetic basis of resistance varieties has seen great progress. For example, 31 quantitative trait loci associated with downy mildew resistance have been described in grapevine with different genetic backgrounds (Koledenkova et al., 2022; VIVC, 2023). Additionally, a set of resistance genes (*R* genes) belonging to the nucleotide‐binding site/leucine‐rich repeat (NBS‐LRR) family, such as *VaRGA1* (Li et al., 2017; Tian et al., 2019), *RGA5* (Fan et al., 2015), *VqCN* (Zhang et al., 2018), *VpRPW8s* (Lai et al., 2018) and a leucine‐rich repeat receptor‐like kinase (LRR‐RLK) family member *VaHAESA* (Liu, Zhang, et al., 2018), have been functionally deciphered. Some *R* genes, such as *MrRPV1*, have been introduced into susceptible grapevine for *P. viticola* resistance (Feechan et al., 2013). Besides the typical R proteins, other resistance‐related proteins, such as transcription factors VvWRKY2 (Mzid et al., 2007), VvWRKY11 (Liu, Yang, et al., 2011), VvWRKY1 (Marchive et al., 2013), MrWRKY30 (Jiang et al., 2015), VvWRKY33 (Merz et al., 2015), MrCBF2 (Wu et al., 2017), pathogenesis‐related proteins VpPR10.1 (Ma et al., 2018; Su et al., 2018), VpPR10.2 (He et al., 2013), VaTLP (He et al., 2017), aldehyde dehydrogenases VpALDH2B4 (Wen et al., 2012), glycoproteins (Guillier et al., 2015) and biomarkers (Batovska et al., 2009), are also involved in downy mildew resistance (Figure 4). However, introgression of these resistance factors into the traditional susceptible cultivars is a difficult project. On the one hand, hybridization between resistant and susceptible species is hampered by their difference in chromosome number. On the other hand, introgression of resistance genes to susceptible species leads to linkage drag of undesired agronomic traits from resistant species that may remain even after successive cycles of backcrossing (Blanc et al., 2012). Moreover, limited understanding of the resistance‐breaking isolates also affects the deployment of resistant varieties in nature. It is therefore challenging work to incorporate resistance durability and maintain important agronomic traits in grapevine breeding (Batovska et al., 2009; Peressotti et al., 2010).

### 5.4 Spray‐induced gene silencing of pathogen genes

The crosstalk of sRNA between plant hosts with their fungal and oomycete pathogens has been investigated in some pathosystems (Brilli et al., 2018; Wang et al., 2016), providing new insight into disease management in crops. For example, external application of double‐stranded (ds)RNA has been developed as a promising tool to protect plants against various pathogens, such as *Fusarium graminearum* (Koch et al., 2016), *Sclerotinia sclerotiorum* (McLoughlin et al., 2018) and *Botrytis cinerea* (Nerva et al., 2020). In grapevine, the application of dsRNA *PvDCL1/2* displayed both protective and curative properties against *P. viticola* (Haile et al., 2021). These tests provide promising tools by which RNA‐based resistant plants or agrochemical alternatives for plant disease management can be developed. However, the mechanism behind the uptake and transport of externally applied dsRNA into host plants remains unclear.

### 5.5 Natural compounds for disease control

Except for the aforementioned measures adopted to control grapevine downy mildew disease, various types of nature or synthetically produced compounds, including carbohydrate polymers, lipids and (glyco)peptides, that exhibit toxic or prohibitive effects on *P. viticola* infection have been developed and applied alone or with other copper‐based formulations to control downy mildew in grapevine (Garde‐Cerdán et al., 2017). Here, we review the inhibitory effects and functional mechanisms of previously characterized compounds.

#### 5.5.1 Laminarin

Laminarin, a natural linear β‐1,3‐glucan oligosaccharide extracted from the brown alga *Laminaria digitata*, deprived of antimicrobial activity, elicits defence in tobacco (Klarzynski et al., 2000), grapevine (Aziz et al., 2003; Gauthier et al., 2014), *Arabidopsis* (Ménard et al., 2004), alfalfa (Cardinale et al., 2000), rice (Inui et al., 1997) and bean (Mithöfer et al., 1999). Defence reactions elicited by laminarin in grapevine cells include calcium influx, alkalinization of the extracellular medium, oxidative burst, activation of mitogen‐activated protein kinases, expression of pathogenesis‐related genes, increase in chitinase and β‐1,3‐glucanase activities, and production of phytoalexins (resveratrol and ε‐viniferin) (Aziz et al., 2003; Gauthier et al., 2014). Although laminarin is able to elicit defence responses in grapevine, protection against *P. viticola* is unsatisfactory, which could result from the low penetration rate of hydrophilic compounds into the leaf, and laminarin acts solely as an elicitor of plant defence rather than as a toxic compound against oomycetes (Paris et al., 2019).

#### 5.5.2 PS3

PS3, a sulphated derivative of laminarin, is considered to be the most efficient polysaccharidic resistance inducer against grapevine downy mildew among the reported elicitors (Héloir et al., 2018). PS3 triggers grapevine resistance via a priming phenomenon in which the compound does not elicit classical early signalling events but triggers an enhanced and prolonged plasma membrane depolarization in grapevine cells and causes much more effective resistance against downy mildew (Chalal et al., 2015; Gauthier et al., 2014). The difference in defence responses triggered by laminarin and PS3 may result from the distinct systems evolved by plants to perceive the two compounds (Ménard et al., 2004). Recently, some reports have claimed that the formulation of resistance inducers plays a critical role in their cuticular diffusion and control efficacy against plant diseases. For example, the penetration efficacy of PS3 through leaf cuticle, stomata, anticlinal cell walls and trichomes can be enhanced by a highly ethoxylated surfactant Dehscofix CO125 (DE) and its content is much higher on the abaxial surface of the leaf than on the adaxial surface, which is helpful to guide its practical use in the field (Paris et al., 2016).

#### 5.5.3 Essential oils

Essential oils (EOs) are another efficient and promising natural protection alternative (Rienth et al., 2019). Terpenes and terpenoids are the main categories of EO compounds and other rare categories including nitrogen‐ and sulphur‐containing compounds, coumarins and homologues of phenylpropanoids (Nazzaro et al., 2017). The antimicrobial activity of EOs might be caused by the properties of terpenes/terpenoids, which are capable of disrupting the cell membrane, causing cell death or inhibiting the sporulation and germination of fungi (Nazzaro et al., 2017; Rienth et al., 2019). A growing amount of evidence indicates that the efficiency of EOs tested in the greenhouse is usually inconsistent with that in the field, which may be attributed to EO degradation caused by light, heat, oxygen, humidity, metal contaminant, application time and poor rain‐fastness (Rienth et al., 2019; Turek & Stintzing, 2013). For example, grapevine treated with sage extract (*Salvia officinalis*) provides a high level of sustained disease control efficacy against *P. viticola*. However, due to the degradation caused by long‐term rainfall, the control efficiency can be significantly reduced in rainy years (Dagostin et al., 2010).

#### 5.5.4 β‐Aminobutyric acid

β‐aminobutyric acid (BABA) has been well known as a resistance inducer to protect a wide range of plant species against biotic and abiotic stresses (Cohen, 2002; Hamiduzzaman et al., 2005; Zimmerli et al., 2008). In penetrated plant cells BABA is thought to block the translocation of nutrients into the haustoria, thereby inhibiting mycelial growth and sporangial production (Hamiduzzaman et al., 2005; Steiner & Schönbeck, 1997). However, BABA‐mediated resistance in plants is most probably based on the priming mechanism rather than direct antimicrobial activities (Conrath et al., 2002; Hamiduzzaman et al., 2005; Ton et al., 2005). In response to *P. viticola*, BABA primes the production of NADPH oxidase‐dependent reactive oxygen species and the deposition of callose and lignin (Dubreuil‐Maurizi et al., 2010; Hamiduzzaman et al., 2005). However, BABA does not elicit typical defence‐related early signalling events such as any variation of cytosolic calcium content, nitric oxide production, reactive oxygen species production, mitogen‐activated protein kinase (MAPK) phosphorylation and defence‐related gene expression in grapevine cells (Dubreuil‐Maurizi et al., 2010).

#### 5.5.5 Chitosan

Chitosan, a totally or partially deacetylated derivative of chitin, confers high protection against grapevine diseases caused by *B. cinerea* and *P. viticola* (Aziz et al., 2006; Romanazzi et al., 2002; Trotel‐Aziz et al., 2006). The polycationic β‐1,4‐linked‐d‐glucosamine polymer forms a semipermeable film that functions as a physical barrier around infection sites, thereby inhibiting pathogens and inducing defence responses in the host tissues (Garde‐Cerdán et al., 2017; Krzyzaniak et al., 2018). It is thought that the activity of chitosan results from its binding to membrane receptors and is dependent on the molecular weight and the degree of *N*‐acetylation (Aziz et al., 2006; Kauss et al., 1989). In grapevine, treatment with chitosan triggers a variety of defence reactions, including the stimulation of lipoxygenase, phenylalanine ammonia‐lyase, chitinase and β‐1,3‐glucanase activities as well as the accumulation of phytoalexins and pathogenesis‐related proteins (Aziz et al., 2006; Trotel‐Aziz et al., 2006).

Other protective compounds, including soybean and casein hydrolysates (Lachhab et al., 2014), glutamate fermentation by‐product (peptidoglycan; Chen et al., 2014), phenolic compounds (preformed gallocatechin derivatives and induced flavonoids; Dai et al., 1995), protein derivatives (Cappelletti et al., 2016), glycyrrhizin (Tröster et al., 2017), benzothiadiazole and fosetyl‐aluminium (Dufour et al., 2016), dehydroeffusol (Thuerig et al., 2016), vitamin B1 (Boubakri et al., 2012), vitamin B2 (Boubakri et al., 2013), *O*‐methylated flavanols and hydroxycinnamic acids (Andreu et al., 2018), larixyl acetate and larixol (Thuerig et al., 2018), also display effective protective efficiency against downy mildew. The compound or its main active constituent can impose direct fungicidal or inhibitory activity (antifungal activity) (Andreu et al., 2018; Boubakri et al., 2012; Dufour et al., 2016) and/or trigger indirect effects including oxidative burst, cytosolic calcium variations, mitogen‐activated protein kinases activation, upregulation of an array of defence response genes, callose and lignin deposition, phytoalexin accumulation, phytohormone production, modification of grapevine phyllosphere microbial communities and hypersensitive response‐like cell death. Some natural products have a dual mode of action (elicitor of grapevine defences and antimicrobial), suggesting their potential as ecofriendly candidates in the control of grapevine downy mildew (Boubakri et al., 2012; Krzyzaniak et al., 2018). Even though some of these compounds exhibit ideal inhibitory activities against *P. viticola*, whether or not these compounds or their active constituents have an effect on the qualitative parameters of grape, must and wine needs further evaluation.

## 6 CONCLUSION

*P. viticola* has become a serious threat to the viticulture globally. A comprehensive insight into the infection strategies and conditions (such as temperature and humidity), pathogenicity mechanism and plant defence response contributes to establish efficient disease management strategies against downy mildew. Decoding the genome of *P. viticola* helps researchers to identify and characterize key pathogenicity‐related genes that potentially serve as chemical fungicide targets. Revealing the pathogen–host interactive regulation also facilitates the matching of avirulence and resistance genes, which provides important genetic resources for disease resistance breeding. Identification of plant‐derived chemical compounds also provides large numbers of valuable and attractive candidates for the development of environmentally friendly fungicides.

Even though great progress in downy mildew control has been made, there is still work to be done to overcome obstacles in plant breeding and disease management. For example, the high risk of *P. viticola* breaking *R* gene‐mediated resistance and the sustainability of resistant grapevine varieties makes it a challenging project to incorporate multiple resistance genes into susceptible species to extend the resistance duration without the loss of desirable phenotypic traits in grapevine breeding. Moreover, appropriate formulations are urgently required to maintain the duration and efficiency of plant‐derived compounds against *P. viticola*.

## CONFLICT OF INTEREST STATEMENT

The authors are not aware of any affiliations, memberships, funding or financial holdings that might be perceived as affecting the objectivity of this review.

## Data Availability

Data sharing is not applicable to this article as no new data were created or analysed.
